# Chronic diseases and emotional disorders are associated with low perception of quality of life in food insecurity/security

**DOI:** 10.3389/fpubh.2022.893601

**Published:** 2022-07-18

**Authors:** Nila Patrícia Freire Pequeno, Natália Louise de Araújo Cabral, Ângelo Giuseppe Roncalli Costa Oliveira, Sandra Patrícia Crispim, Cecília Rocha, Dirce Maria Marchioni, Severina Carla Vieira Cunha Lima, Clélia de Oliveira Lyra

**Affiliations:** ^1^Postgraduate Program in Public Health, Federal University of Rio Grande do Norte, Natal, Brazil; ^2^Department of Nutrition, Federal University of Rio Grande do Norte, Natal, Brazil; ^3^Federal Institute of Education, Science and Technology of Sertão Pernambucano, Petrolina, Brazil; ^4^Department of Nutrition, Federal University of Paraná, Curitiba, Brazil; ^5^School of Nutrition, Centre for Studies in Food Security, Ryerson University, Toronto, ON, Canada; ^6^Department of Nutrition, School of Public Health, University of São Paulo, São Paulo, Brazil

**Keywords:** food security, quality of life, chronic disease, emotional disorders, depression, adults, older adults

## Abstract

Understanding individual perception of Quality of Life (QoL) can help combat social and health inequalities. We aimed to identify factors associated with Low Perceived Quality of Life (LPQoL) in 295 adults and older adults with food security and food insecurity, in the city of Natal, Brazil. A cross-sectional study was conducted from June to December 2019, with collection of data on socioeconomic demographic status, lifestyle information, non-communicable diseases (NCDs) and risk factors, emotional disorders, food (in) security and quality of life. To assess food insecurity, the Brazilian Scale of Food Insecurity—EBIA was used, and the WHOQOL-Bref questionnaire was used to assess quality of life. Poisson's Regression was used to verify associations between variables and LPQoL, stratifying the sample into food secure (FS) and food insecure (FI) groups. In the FI group, being overweight, older adult, having no partner, drinking alcoholic beverages twice a week or more, and not having daily availability of water were associated with LPQoL, and in the FS group, having diabetes, monthly family income in the 1st and 2nd tertiles, and never having studied was associated with LPQoL. Reporting emotional disorders and sleeping < 7 h/day were associated with LPQoL in both FI and FS groups. LPQoL was associated with the occurrence of NCDs and risk factors, and emotional disorders, regardless of the food security measure. However, the lack of adequate access to water highlights the social vulnerability of the FI group.

## Introduction

Access to food, directly linked to improved living conditions for populations, is one of the current challenges in the face of increasing threats to food security (FS). FS is defined *as the realization of the right of everyone to regular and permanent access to quality food, in sufficient quantity, without compromising access to other essential needs, based on health-promoting dietary practices that respect cultural diversity and are environmentally, culturally, economically, and socially sustainable* (1). Such challenges include climate change, territorial conflicts, obesity and malnutrition pandemics, and more recently, infectious disease pandemics such as COVID-19 (2, 3). Current food systems have greatly improved human health over the past century, helping to increase food security and life expectancy, yet paradoxically, these same food systems have become a major contributor to global epidemics of chronic non-communicable diseases (NCDs) from the spread of nutritionally inadequate diets (2).

Food insecurity (FI) comprises the lack of access to adequate food, predominantly due to a low socioeconomic condition—low income, low education, unemployment, lack of access to basic sanitation, etc. (4), resulting from poverty, health problems, and suboptimal food management strategies (5).

Access to food is an important factor for Quality of Life (QoL), and deprivation of food, in quantity and/or quality, malnutrition, and nutritional deficiencies are associated with food insecurity (5, 6). Thus, research has also evidenced the association of food insecurity with non-communicable diseases (NCDs) and their risk factors, with a higher prevalence found in more economically poor populations (6, 7). This confirms that food limitation, hunger, and nutritional deficiencies are not the only manifestations of FI (7). Mental illness, mood disturbance, and depressive symptoms have also been associated with food insecurity (8).

In this sense, FI is one of the conditions that can affect QoL, defined as individuals' perception of their position in life in the cultural and value context in which they live and concerning their goals, expectations, standards, and concerns (9). FI can pose not only a physical threat, but a strong psychosocial stressor to the individual and can increase the risk of poor overall self-perceived physical and mental health, such as developing worry and anxiety, feelings of exclusion, deprivation and alienation, distress, and adverse family and social interactions (5). In addition, QoL scores and their domains (physical, functional, social, and emotional wellbeing) decrease significantly with increasing FI (5, 10).

In countries like Brazil, with 36.7% of its households or 84.9 million inhabitants in some degree of food insecurity (11), studies that evaluate how the physical, psychological, and social dimensions that FI affects the QoL of populations, can assist in the planning, implementation, and/or better evaluation of public policies directed at addressing social and health inequalities. Still, studies that conduct a comprehensive assessment of QoL in adult and elderly populations in Brazil are scarce (12), especially in the Northeast region of the country, one of the poorest and with the worst social indicators. This lack of information hinders the comparison between regional/national and international surveys, and the estimates of quality of life parameters. In this sense, the hypothesis of our study is that low quality of life is more frequent in households with food insecurity. Thus, this article aims to identify the factors associated with low perceived QoL in adults and older adults with FI and FS in a population of a capital city in the Northeast of Brazil.

## Methods

### Study design and population

This is a cross-sectional, analytical, and exploratory study, using a convenience sub-sample of the BRAZUCA—Brazilian Usual Consumption Assessment survey, developed among five public Brazilian universities, with the University of São Paulo—USP, as the coordinating center. The data presented derive from the research “Food insecurity, health, and nutrition conditions in adult and older adult population of a capital city in the Northeast of Brazil: BRAZUCA Natal Study”, developed by the Nutrition Department of the Federal University of Rio Grande do Norte (UFRN).

The survey involved a complex sampling plan, considering a probabilistic sample by conglomerates in two stages (census sectors and households). It drew 66 census sectors of the municipality of Natal-RN and their households, with probability proportional to size (number of domiciles), ordered, before the drawing, according to schooling indicators (demographic census of 2010). For the survey, up to two residents from different strata (women aged 20–59, women aged 60 and over, men aged 20–59, or men aged 60 and over) were selected in each household. Only those individuals who were informed about the objectives, risks, and benefits, and who agreed to participate in the study by signing the Informed Consent Form (ICF), participated in the study.

In this paper, data from 295 participants interviewed, both male and female, were evaluated during the period from June to December 2019.

The research was approved by the Research Ethics Committee of the Onofre Lopes University Hospital of UFRN (no. 96294718.4.2001.5292).

### Data collection

The interviews were conducted at home or in health centers, using a questionnaire developed on a digital platform (https://five.epicollect.net/), applied using smartphones or tablets, containing information about the dependent variables (food safety/food insecurity and QoL) and the independent variables (socioeconomic, demographic, lifestyle, and health conditions). Anthropometric measurements were also collected at homes or health centers, and the equipment was taken to these locations. All interviewers were trained, and support manuals were made available regarding the techniques of anthropometric measurements collection and how to fill out the electronic questionnaire. The weight of the interviewees was checked using an electronic scale with a capacity of 150 kg and precision of 50 g, and height was measured using a portable stadiometer with a precision of 1.0 mm and a non-slip base.

### Food security

The state of food security was assessed by the Brazilian Scale of Food Insecurity—EBIA (in Portuguese), nationally validated (13) and adopted by the Brazilian government in population surveys such as the National Household Sample Survey (PNAD in Portuguese) and the Family Budget Survey (POF in Portuguese). The objective of the EBIA is to verify the perception and experience of hunger within the household, as well as the difficulty in accessing food (13). It is subdivided into four levels: food security (FS), mild, moderate, and severe food insecurity (FI), which portray concerns about access to food, as well as quantitative reduction of food consumption within the household, among adults or to a more severe degree, among children.

### Quality of life assessment

QoL was assessed using the WHOQOL-Bref instrument from the WHO, translated, and validated for the Brazilian population (14). The instrument is divided into 26 questions, being 2 general questions and 24 divided into four domains that analyze different aspects of QoL, measured in scores ranging from 0 to 100: physical (pain and discomfort, energy and fatigue, sleep and rest, mobility, activities of daily living, dependence on medication or treatment, and ability to work), psychological (positive feelings, thinking, learning, memory and concentration, self-esteem, body image and appearance, negative feelings, spirituality, religiosity, and personal beliefs), social relationships (personal relationships, social support, and sexual activity), and environment (availability and quality of physical safety and security, home environment, financial resources, and health and social care), opportunity to acquire new information and skills, participation in/and opportunities for recreation or leisure, physical environment (pollution, noise, traffic, weather, transportation). Higher scores indicate better QoL. Specific syntax (14) developed by WHO was used to calculate the scores for each domain and analyzed in the Statistical Package for the Social Science (SPSS) Statistics version 25.

### Socioeconomic and demographic characteristics

The variables evaluated were as follows: sex (men, women), age (20–39 years, 40–59 years, ≥60 years), race/skin color (white, non-white), education (never studied, 1–8 years, 9–11 years, ≥12 years), civil status (with a partner, without a partner), monthly family income in tertiles (1st ≤ US$ 347.0, 2nd - US$ 347.0–620.0, 3rd ≥ US$ 620.0), number of residents in the household (<3, 4–5, ≥6), households with children under 18 (no, yes), employment condition (yes, retired/pensioner, no), number of rooms in the household (≥6 or more, <6), daily availability of water in the household (yes, no), water used for drinking (mineral or treated at home, untreated), sanitary sewage (sanitary sewage, septic tank, rudimentary tank or ditch), destination given to garbage (collected by urban cleaning service with frequency ≥ 3 times/week, placed in a dumpster). For the conversion of the Brazilian real to US dollars, an investigation was carried out on the exchange rate on December 31, 2019.

### Lifestyle

The following were assessed: physical activity (active/very active, irregularly active, sedentary) by the International Physical Activity Questionnaire—IPAQ (15), alcohol consumption (never, 1–4 times per month, ≥2 times/week), tobacco consumption—is/was a smoker (no, yes), and sleep duration (≥7 h, <7 h).

### Health and nutrition

Self-reported health conditions were analyzed using the questions “Do you have hypertension (high blood pressure)?” (No, yes), “Do you have diabetes? (No, yes),” “Do you have depression/anxiety/emotional disorders?” (No, yes).

Anthropometric nutritional status was assessed using BMI, with weight and height measured. For the classification of the Body Mass Index—BMI, we used the World Health Organization—WHO classification (16) for adults and the Lipschitz classification (17) for the elderly (considering the changes in body composition resulting from aging). For analysis purposes, for adults and older adults, the variable overweight (yes, no) was considered.

### Statistical analysis

Descriptive analyses were performed to identify prevalence percentages and confidence intervals (95%) of the variables studied. To verify differences in the distribution of scores of the QoL domains concerning food security/insecurity status, Kruskal-Wallis statistical analysis was performed. The characterization of the study population was stratified into two groups: Food Secure (FS) and Food Insecure (FI—all levels). Pearson's χ^2^ test was used to verify the association between FS/FI and socioeconomic, demographic, lifestyle, and health variables.

To verify the association between the dependent variables “FS and FI” and “QoL” with the independent variables, Poisson regression was used, with a robust estimator, aiming to identify the crude and adjusted prevalence ratios (PR), besides controlling for confounding variables. After the bivariate analysis, the independent variables with <20% association (*p* < 0.20) entered the multivariate analysis and only the variables with a 5% significance level (*p* < 0.05) remained in the final model. Considering that QoL and food insecurity can be influenced by gender and age, we chose to keep them in the final model, regardless of statistical significance. In addition, the interaction between emotional disorders and sleep duration was tested earlier and included to adjust the final model.

To verify the association of independent variables with Low Perceived QoL (LPQoL) in the FS and FI groups, the QoL domains were categorized according to the population median score identified in each domain. Thus, individuals with scores above the median were identified with good QoL perception, and individuals with scores below the median were identified with low QoL perception (LPQoL). Therefore, individuals with scores below the median in each QoL domain were considered for the analysis. The reason for stratifying the analyzes according to the food security/insecurity situation was based on the literature, which addresses differences between factors associated with poor quality of life in populations with FI and FS. Individuals with FI generally have worse socioeconomic and health conditions than individuals with FS (18–20), which exposes them to a situation of greater social vulnerability and differentiated risk. Individuals with FI have lower quality of life scores than those with SF, especially in aspects related to socioeconomic conditions, such as income, schooling and marital status, chronic non-communicable diseases, physical and mental health (8, 21, 22).

## Results

Food insecurity was observed in 48.5% of the interviewees (*n* = 143), being associated with the following variables: sex, age, schooling, monthly family income, employment condition, number of residents in the household, number of rooms, presence of children under 18 in the household, daily availability of water in the household, water used for drinking, sanitary sewage, and overweight (*p* < 0.05) (Table 1).

**Table 1:** Socioeconomic, demographic, lifestyle, and health characteristics of adults and older adults according to food security status.

**Variables/categories**	**Food insecurity (*****n*** = **143)**		**Food security** ***(n*** = **152)**		***p*** ^*^
	**%**	**95% CI** ^a^	**%**	**95% CI** ^a^	
**Sex**					<0.001
Men	32.9	25.3–41.2	48.7	40.5–56.9	
Women	67.1	58.8–74.8	51.3	43.1–59.5	
**Age group (years)**					<0.001
20–39	30.1	22.7–38.3	15.8	10.4–22.6	
40–59	33.6	25.9–41.9	29.6	22.5–37.5	
≥60	36.4	28.5–44.8	54.6	46.3–62.7	
**Civil status**					0.317
With a partner	60.8	52.3–68.9	66.4	58.4–73.9	
Without a partner	39.2	31.1–47.7	33.6	26.1–41.7	
**Schooling (full years)**					<0.001
≥12	3.5	1.1–8.0	20.5	14.4–27.9	
9–11	32.9	25.3–41.2	32.5	25.1–40.5	
1–8	52.4	43.9–60.9	41.7	33.8–50.0	
Never studied	11.2	6.5–17.5	5.3	2.3–10.2	
**Monthly family income in tertiles^b^**					<0.001
3rd tertil (≥US$ 620.0)	22.9	16.2–30.7	54.3	45.7–62.9	
2nd tertil (US$ 347.0–620.0)	34.3	26.5–42.8	23.9	17.1–31.9	
1st tertil ( ≤ US$ 347.0)	42.9	34.5–51.5	21.7	15.2–29.6	
**Employment condition**					<0.013
Yes	28.4	21.1–36.6	31.5	24.2–39.7	
Retired/pensioner	28.4	21.1–36.6	40.9	33.0–49.3	
No	43.3	35.0–51.9	27.5	20.5–35.4	
**Number of residents in household**					<0.001
<3	44.1	35.8–52.6	63.8	55.6–71.4	
4–5	35.0	27.2–43.4	32.2	24.9–40.3	
≥6	21.0	14.6–28.6	3.9	1.5–8.4	
**Number of rooms in the household**					<0.008
≥6	65.0	56.6–72.8	78.9	71.6–85.1	
<6	35.0	27.2–43.4	21.1	14.9–28.4	
**Households with children under 18**					<0.001
No	44.8	36.4–53.3	73.7	65.9–80.5	
Yes	55.2	46.7–63.6	26.3	19.5–34.1	
**Daily availability of water in the household**					0.012
Yes	70.6	62.4–77.9	82.9	76.0–88.5	
No	29.4	22.1–37.6	17.1	11.5–24.1	
**Water used for drinking**					<0.001
Mineral or treated at home	79.0	71.4–85.4	94.7	89.9–97.7	
No treatment at home	21.0	14.6–28.6	5.3	2.3–10.1	
**Sanitary sewage system**					<0.002
Sanitary sewage	44.8	36.4–53.3	66.4	58.4–73.9	
Septic tank	50.3	41.9–58.8	30.3	23.1–38.2	
Rudimentary	4.9	2.0–9.8	3.3	1.1–7.5	
**Consumption of alcoholic beverages**					0.505
Never	65.7	57.3–73.5	60.5	52.3–68.4	
1–4 times a month	24.5	17.7–32.4	25.7	18.9–33.4	
≥ 2 times a week	9.8	5.5–15.9	13.8	8.8–20.3	
**Physical activity**					0.833
Active/very active	46.5	38.1–55.0	50.0	41.8–58.2	
Irregularly active	35.9	28.0–44.4	33.6	26.1–41.7	
Sedentary	17.6	11.7–24.9	16.4	10.9–23.3	
**Sleep duration (hours)**					0.593
≥7	61.3	52.6–69.5	64.4	56.0–72.1	
<7	38.7	30.5–47.4	35.6	27.9–44.0	
**Emotional disorders (depression. anxiety, etc.)**					0.090
No	63.1	54.6–71.1	72.4	65.5–79.3	
Yes	36.9	28.9–45.4	27.6	20.7–35.5	
**Hypertension**					0.488
No	63.3	54.7–71.3	59.3	51.0–67.3	
Yes	36.7	28.7–45.3	40.7	32.7–49.0	
**Diabetes mellitus**					0.893
No	80.7	73.2–86.9	81.3	74.2–87.2	
Yes	19.3	13.1–26.8	18.7	12.8–25.8	
**Overweight**					0.046
No	23.6	16.8–31.5	34.2	26.7–42.4	
Yes	76.4	68.5–83.2	65.8	57.6–73.3	

*Brazuca Natal Study (n = 295)*.

a *95%CI: 95% confidence intervals*.

b *Approximate values in American dollars, after conversion of the Brazilian Real, on December 31st/2019*.

* *Pearson's chi-square (χ^2^) test*.

**Figure 1:**
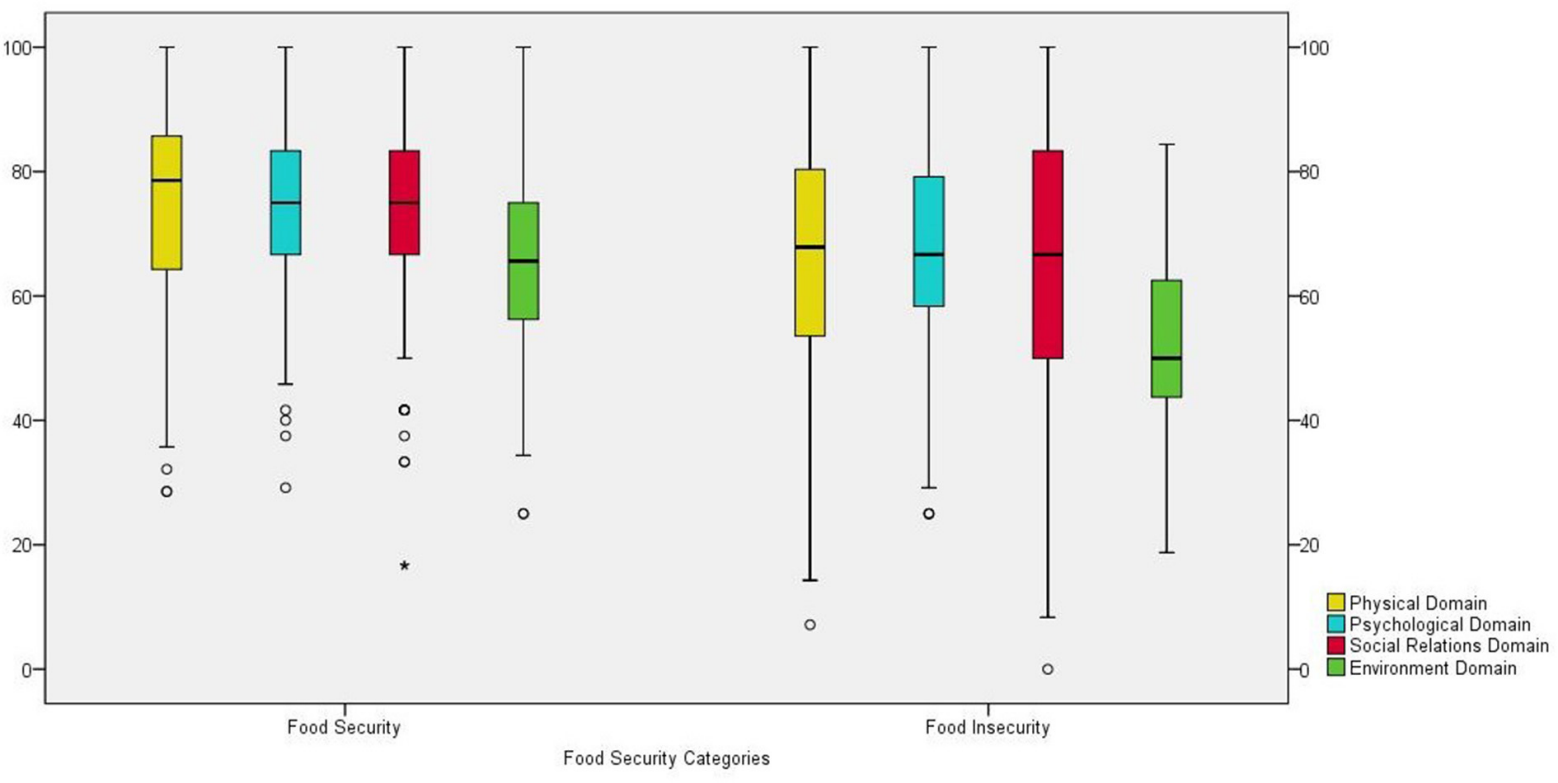
Boxplot of stratified analysis of groups Food Secure and Food Insecure (Md; IIQ) by scores of the domains of Quality of Life (QoL) of adults and older adults. Study Brazuca Natal (*n* = 295).

In the QoL assessment of the total population (*n* = 295), median scores were 71.4 (Q1 60.7; Q3 85.7) for the “physical domain”; 70.8 (Q1 62.5; Q3 83.3) for the “psychological domain”; 75.0 (Q1 58.3; Q3 83.3) for the “social relations” domain, and 59.4 (Q1 46.9; Q3 68.8) for the “environmental domain” (data not shown in table). In the stratified analysis in “food secure” and “food insecure” groups, we observed higher scores in all QoL domains in the group with food security. The Environment domain showed the lowest median in both groups, but much lower in the food insecure group (Figure 1).

Regarding the perception of QoL in the FS group and associated variables, it was observed in the multivariate analysis that “never studied” increased by 2.51 and 4.33 times the probability of presenting a LPQoL in the domains of social relations and environment, respectively. Having family income in the 1st and 2nd tertiles increased by 2.03 and 1.92 times the probability of a LPQoL in the environment domain. Reporting emotional disorders (PR = 2.55; 95% CI 1.77–3.68) in the physical domain; emotional disorders x sleep <7 h/day interaction (PR = 3.32; 95% CI 2.25–4.91) in the psychological domain; sleeping <7 h/day (PR = 2.06; 95% CI 1.32–3.20) and (PR = 1.61; 95% CI 1.08–2.41) in the social relations and environment domains, respectively; having diabetes (PR = 1.68; 95% CI 1.15–2.45) in the physical domain, were also associated with lower QoL scores (Table 2).

**Table 2:** Crude and adjusted Prevalence Ratios (PR) and Confidence Intervals (95%) of the variables associated with low perceived quality of life^*^ (LPQoL) in the physical, psychological, social relations and environment domains of adults and older adults with Food Security (FS).

**Variables/categories**	**Physical (*****n*** = **59)**		**Psychological (*****n*** = **46)**		**Social relations (*****n*** = **53)**		**Environment (*****n*** = **57)**	
	**Crude PR**	**Adjusted PR** ^a^	**Crude PR**	**Adjusted PR** ^a^	**Crude PR**	**Adjusted PR** ^a^	**Crude PR**	**Adjusted PR** ^a^
	**(95% CI)**	**(95% CI)**	**(95% CI)**	**(95% CI)**	**(95% CI)**	**(95% CI)**	**(95% CI)**	**(95% CI)**
**Age group (years)**								
20–39	1.00		1.00	1.00	1.00	1.00	1.00	1.00
40–59	1.31 (0.54–3.16)	–	1.44 (0.61–3.41)	0.89 (0.48–1.68)	2.62 (0.63–10.86)	1.51 (0.64–3.57)	1.64 (0.79–3.37)	1.07 (0.62–1.87)
≥60	1.45 (0.64–3.24)	–	0.87 (0.36–2.10)	0.60 (0.31–1.16)	2.35 (0.59–9.36)	1.19 (0.48–2.94)	0.97 (0.45–2.06)	0.53 (0.28–0.99)
**Schooling (full years)**								
≥12	1.00	1.00	1.00		1.00	1.00	1.00	1.00
9–11	1.43 (0.64–3.16)	1.73 (0.99–3.03)	1.07 (0.45–2.53)	–	0.71 (0.16–3.18)	0.91 (0.45–1.83)	2.62 (0.84–8.19)	2.29 (0.76–6.94)
1–8	1.26 (0.57–2.77)	1.46 (0.83–2.56)	0.99 (0.43–2.28)	–	2.70 (0.89–8.23)	1.52 (0.82–2.83)	3.42 (1.15–10.18)	2.49 (0.78–7.90)
Never studied	2.00 (0.75–5.33)	2.22 (0.89–5.54)	1.33 (0.38–4.73)	–	2.67 (0.60–11.92)	2.51 (1.10–5.71)	5.33 (1.72–16.54)	4.33 (1.32–14.27)
**Monthly family income in tertiles^b^**								
3rd tertil (≥US$ 620.0)	1.00		1.00		1.00		1.00	1.00
2nd tertil (US$ 347.0–620.0)	1.24 (0.68–2.26)	–	1.16 (0.59–2.31)	–	1.63 (0.76–3.49)	–	2.41 (1.36–4.28)	1.92 (1.15–3.21)
1st tertil ( ≤ US$ 347.0)	1.38 (0.73–2.63)	–	0.99 (0.42–2.33)	–	1.11 (0.40–3.06)	–	2.07 (1.07–4.02)	2.03 (1.17–3.52)
**Sanitary sewage system**								
Sanitary sewage	1.00		1.00	1.00	1.00		1.00	
Septic Tank	0.94 (0.54–1.67)	–	1.70 (0.92–3.14)	1.62 (1.04–2.53)	0,74 (0.34–1.61)	–	1.10 (0.65–1.87)	–
Rudimentary	1.56 (0.71–3.45)	–	0.80 (0.13–4.91)	1.00 (0.17–6.02)	0.69 (0.11–4.21)	–	1.56 (0.71–3.45)	–
**Consumption of alcoholic beverages**								
Never	1.00		1.00		1.00		1.00	1.00
1–4 times a month	0.76 (0.40–1.45	–	0.58 (0.24–1.37)	–	0.89 (0.39–2.03)	–	0.46 (0.22–0.97)	0.53 (0.24–1.18)
≥2 times a week	0.71 (0.32–1.57)	–	0.87 (0.38–1.96)	–	0.67 (0.22–2.04)	–	0.46 (0.19–1.12)	0.48 (0.25–0.91)
^*^Emotional disorders								
No	1.00	1.00	1.00		1.00		1.00	
Yes	2.77 (1.47–4.52)	2.55 (1.77–3.68)	3.61 (1.96–6.64)	–	1.02 (0.48–2.19)	–	1.12 (0.67–1.88)	–
**Sleep duration (hours)**								
≥7	1.00		1.00		1.00	1.00	1.00	1.00
<7	1.83 (1.10–3.03)	–	1.99 (1.09–3.66)	–	2.25 (1.11–4.54)	2.06 (1.32–3.20)	1.47 (0.91–2.38)	1.61 (1.08–2.41)
^**^Interaction emotional disorders x sleep (hours)								
No ≥ 7	1.00		1.00	1.00	1.00		1.00	
Yes <7	2.37 (1.53–3.67)	–	3.29 (1.99–5.44)	3.32 (2.25–4.91)	1.19 (0.93–1.52)	–	1.07 (0.84–1.37)	–
**Diabetes mellitus**								
No	1.00	1.00	1.00		1.00		1.00	
Yes	1.63 (0.93–2.85)	1.68 (1.15–2.45)	1.08 (0.46–2.58)	–	1.37 (0.56–3.33)	–	1.79 (1.10–2.94)	-

*Brazuca Natal Study (n = 152)*.

PR, Prevalence ratios. Crude and adjusted PR by Poisson regression, with robust estimator (p < 0.05). Adjusted PR for all variables in the table and sex. ^*^Low perceived of QoL: Physical Domain: <71.4; Psychological Domain: <70.8; Social relations domain: <75.0; Environment Domain: <59.4.

* *This variable was not included in the statistical model, considering the Psychological domain as a dependent variable*.

** *This variable was not included in the statistical model, considering the Physical domain as a dependent variable*.

a *Values not shown for the Adjusted Prevalence Ratio were not included in the statistical model*.

b * Approximate values in American dollars, after conversion of the Brazilian Real, on December 31st/2019*.

In the FI group, not having a partner increased the odds of having LPQoL in the physical, psychological, and social relationships domains by 1.60, 1.73, and 2.38 times, respectively, while having emotional disorders increased the odds of having LPQoL by 1.68, 1.48, and 1.48 times in the same domains. Being overweight and consuming alcoholic beverages ≥ 2 times per week increased the likelihood of LPQoL in the environment domain by 1.41 and 1.30 times, respectively, while sleeping <7 h/day increased the same likelihood in the psychological domain by 1.65 times. Being ≥ 60 years old showed a higher probability of LPQoL in the physical and psychological domains compared to young adults (PR = 2.74; 95% CI 1.71–4.40) and (PR = 2.04; 95% CI 1.27–3.29). Not having daily availability of water in the household increased by 1.25 times the risk of having LPQol in the environmental domain, while drinking untreated water at home increased 1.70 times the probability of having LpQol, in the assessment of the psychological domain (Table 3).

**Table 3:** Crude and adjusted Prevalence Ratios (PR) and Confidence Intervals (95%) of the variables associated with low perceived quality of life (LPQoL) in the physical, psychological, social relations and environment domains of adults and older adults with Food Insecurity (FI).

**Variables/categories**	**Physical (*****n*** = **75)**		**Psychological (*****n*** = **73)**		**Social relations (*****n*** = **72)**		**Environment (*****n*** = **103)**	
	**Crude PR**	**Adjusted PR** ^a^	**Crude PR**	**Adjusted PR** ^a^	**Crude PR**	**Adjusted PR** ^a^	**Crude PR**	**Adjusted PR** ^a^
	**(95% CI)**	**(95% CI)**	**(95% CI)**	**(95% CI)**	**(95% CI)**	**(95% CI)**	**(95% CI)**	**(95% CI)**
**Sex**								
Men	1.00		1.00		1.00	1.00	1.00	1.00
Women	1.16 (0.72–1.88)	–	1.51 (0.89–2.59)	–	0.88 (0.54–1.42)	0.65 (0.43–0.98)	0.98 (0.75–1.28)	1.08 (0.84–1.39)
**Age group (years)**								
20–39	1.00	1.00	1.00	1.00	1.00		1.00	1.00
40–59	1.42 (0.74–2.72)	1.50 (0.89–2.52)	0.85 (0.53–1.36)	1.67 (0.98–2.85)	0.55 (0.31–0.99)	–	0.99 (0.74–1.32)	1.05 (0.83–1.34)
≥60	2.18 (1.21–3.92)	2.74 (1.71–4.40)	0.82 (0.50–1.35)	2.04 (1.27–3.29)	0.82 (0.50–1.34)	–	0.93 (0.68–2.28)	1.04 (0.76–1.43)
**Civil status**								
With a partner	1.00	1.00	1.00	1.00	1.00	1.00	1.00	1.00
Without a partner	1.74 (1.17–2.58)	1.60 (1.12–2.28)	1.97 (1.34–2.91)	1.73 (1.23–2.42)	2.22 (1.41–3.48)	2.38 (1.54–3.67)	1.22 (0.96–1.54)	0.98 (0.79–1.21)
**Monthly family income in tertiles^b^**								
3rd tertil (≥US$ 620.0)	1.00		1.00		1.00	1.00	1.00	
2nd tertil (US$ 347.0–620.0)	0.93 (0.62.1.39)	–	0.79 (0.55–1.15)	–	0.76 (0.47–1.24)	0.82 (0.51–1.32)	1.20 (0.80–1.82)	–
1st tertil ( ≤ US$ 347.0)	0.89 (0.60–1.32)	–	0.64 (0.43–0.94)	–	0.59 (0.35–0.98)	0.57 (0.37–0.87)	1.32 (0.89–1.94)	–
**Number of rooms**								
≥6	1.00		1.00		1.00		1.00	1.00
<6	0.83 (0.53–1.32)	–	0.89 (0.58–1.39)	–	1.10 (0.69–1.76)	–	1.26 (0.99–1.58)	1.32 (1.09–1.60)
**Daily availability of water in the household**								
Yes	1.00		1.00		1.00		1.00	1.00
No	1.11 (0.74–1.70)	–	0.98 (0.64–1.51)	–	1.22 (0.77–1.93)	–	1.26 (0.99–1.58)	1.25 (1.02–1.53)
**Water used for drinking**								
Mineral or treated at home	1.00	1.00	1.00		1.00		1.00	
No treatment at home	1.51 (1.00–2.28)	1.70 (1.14–2.55)	1.30 (0.83–2.03)	–	1.35 (0.82–2.25)	–	1.03 (0.75–1.41)	–
**Consumption of alcoholic beverages**								
Never	1.00	1.00	1.00		1.00		1.00	1.00
1–4 times a month	0.62 (0.32–1.19)	1.02 (0.51–2.05)	0.91 (0.54–1.53)	–	1.04 (0.61–1.79)	–	0.94 (0.68–1.30)	0.98 (0.78–1.24)
≥2 times a week	1.51 (0.99–2.30)	1.48 (0.86–2.55)	1.33 (0.79–2.25)	–	1.02 (0.47–2.23)	–	1.23 (0.93–1.62)	1.30 (1.05–1.61)
**Sleep duration (hours)**								
≥7	1.00	1.00	1.00	1.00	1.00		1.00	
<7	1.58 (1.06–2.36)	1.24 (0.85–1.79)	1.52 (1.03–2.24)	1.65 (1.10–2.47)	1.24 (0.79–1.95)	–	1.11 (0.87–1.41)	–
**Emotional disorders (depression, anxiety, etc.)**								
No	1.00	1.00	1.00	1.00	1.00	1.00	1.00	1.00
Yes	1.59 (1.07–2.36)	1.68 (1.19–2.36)	1.62 (1.18–2.23)	1.48 (1.03–2.12)	1.82 (1.17–2.84)	1.48 (1.02–2.15)	1.37 (1.09–1.72)	1.14 (0.93–1.39)
**Overweight**								
No	1.00		1.00		1.00		1.00	1.00
Yes	1.11 (0.68–1.81)	–	1.14 (0.70–1.86)	–	0.95 (0.57–1.58)	–	1.47 (1.00–2.17)	1.41 (1.03–1.92)

*Brazuca Natal Study (n = 143)*.

PR, Prevalence ratios. Crude and adjusted PR by Poisson regression, with robust estimator (p < 0.05). Adjusted PR for all variables in the table and sex. *Low perceived of QoL: Physical Domain: <71.4; Psychological Domain: <70.8; Social relations domain: <75.0; Environment Domain: <59.4.

a *Values not shown for the Adjusted Prevalence Ratio were not included in the statistical model*.

b *Approximate values in American dollars, after conversion of the Brazilian Real, on December 31st/2019*.

## Discussion

The results of this study evidenced the presence of lower scores in all QoL domains in the group with food insecurity. Risk associations between chronic non-communicable diseases and/or risk factors, emotional disorders, and low perceived QoL were identified in both groups, FS and FI.

The literature reports lower QoL scores in food insecure groups (8, 21, 22), pointing to the association of NCDs and their risk factors (such as diabetes, hypertension, obesity, cancer, physical inactivity, smoking, excessive alcohol consumption, emotional disorders, respiratory, neurological, and other chronic problems) with lower scores in the physical and/or mental domains (23, 24). Thus, higher scores in the physical domain of QoL are associated with the absence of NCDs and higher levels of physical activity (23). The results of our study corroborate the literature and indicate the association of NCDs and their risk factors (diabetes, overweight, and alcohol consumption ≥ 2 times per week), and emotional disorders (depression and anxiety), not only in the physical and psychological domains but also in the social relationships and environment domains, revealing a greater impairment of QoL-related aspects in the assessed population.

Individuals who have NCD are more likely to limit daily activities, for presenting, in most cases, physical symptoms such as pain and discomfort, which can reduce functional capacity, reflecting negatively on QoL, especially in the physical domain. On the other hand, QoL is influenced by age, and it is perceived that QoL scores decrease significantly as age increases (25). Age can affect the ability to perform physical exercise and daily activities, through low-quality food intake, nutritional deficiencies, or even malnutrition of the individual (8). This may explain the findings in our study, as being ≥ 60 years old was associated with low perceived QoL in the group with FI. Another issue is the accessibility of food, which can occur for different issues depending on the life cycle. Elderly people report (21, 26) having more difficulty walking long distances to shop, carry groceries, or carry heavy bags, while younger adults report lack of money as a limiting cause to access food. Russell et al. (21) suggest that poor physical functionality can be a major limitation for older people to acquire or prepare food appropriately.

Depressed adults may feel unable to work or generate income and lack the motivation or energy to purchase or prepare food (21). On the other hand, relationships between social inequity and mental health are frequently described in the literature (8, 27), associating the presence of depression, anxiety, and other mental disturbances with lower QoL scores in poor, unemployed, and/or food insecure populations, women, the elderly, and individuals with low education. Low income can lead to negative attitudes toward life, guilt, shame, helplessness, hopelessness, affect mental health, and generate a vicious circle, which can also lead to depressive symptoms (24). A study by Nagata et al. (28), using representative data of 14,786 young American adults aged 24–32 years from Wave IV (2008) of the National Longitudinal Study of Adolescent to Adult Health, revealed associations between FI and mental health among young adults, even with adjustment for confounding variables such as socioeconomic ones, suggesting an independent association (28). Corroborating with the literature, we found that the variable “emotional disorders” was associated with low perceived QoL in both groups (FS and FI). However, in the group with FI, this variable was associated with more QoL domains (physical, psychological, and social relations), while in the FS group it was associated only with the psychological domain when interacting with the sleep variable. We also observed that overweight was associated with low perceived QoL (environment domain) only in the group with FI.

Inadequate sleep (<7 h/day) (29) can lead to depressive symptoms in socially vulnerable individuals, such as those with FI (30). Not having healthy sleep is associated with higher rates of mortality, diabetes, hypertension, coronary heart disease, depression, and traffic accidents (31, 32). Poor quality sleep harms the QoL of healthy and sick people, especially in the physical domain and in self-assessment of health and dissatisfaction with life (31, 32). Our study reinforces the findings in the literature by detecting a high association in the interaction between emotional disorders vs. sleep with low perceived QoL among people who presented FS, and other associations of sleep and emotional disorders (no interaction) in both groups (FS and FI), regardless of age and gender.

Researches has linked FI to inadequate diet quality. Chung et al. (8) using data from the Korea National Health and Nutritional Examination Survey (2012–2013) among 5,862 Koreans aged 20–64 years, founded a greater proportion of food-insecure participants were nutritionally deficient compared with expectations of the 2015 Korean Dietary Reference Intakes, and a significantly adverse mental health status particularly in the *food-insecure household with hunger* group. The study concluded that food insecurity may be significantly associated with adverse mental health indicators and decreased QOL in young/middle-aged Koreans. Russel et al. (21) using data from Blue Mountains Eye Study, an Australian cohort study of community-living individuals aged 49 years and over, with 2,642 participants, also found evidence of associations between reduced physical and mental health and food insecurity and poor diet quality. Despite the results of these surveys, apparently the association between FI and QoL is complex, composed of multi-lateral factors, which increases the debate whether FI status is a predictor or result of health problems and/or diet quality/quantity. As such, FI may compromise some factors of QoL in populations (8). In our study, we were not able to investigate dietary intake between groups; however, we observed a multifactorial association between FI and QoL. The results of the two groups (FS and FI) were similar in the presence of NCD/risk factors and emotional disorders that were associated with low perceived QoL, reflecting the global trend of changing epidemiological and nutritional profiles of adults and the elderly (33).

In the FI group, aspects related to emotional/social support (absence of a partner or spouse) and basic needs for life maintenance and comfort of the living environment (not having daily availability of water and drinking untreated water) were associated with low perceived QoL. Studies indicate that the presence of a partner is associated with better mental health and better perceived QoL, especially in the physical and mental domains, emotional and social support, and can positively assist family socioeconomic status (34, 35). A study conducted with 1,492 Dutch people aged 50 years or more observed that Participants who were married or cohabited scored higher in quality-of-life domains, mainly concerned with the psychological and social domains (34). Another population-based survey study, using data from 12,423 Brazilians aged ≥ 20 years, also observed that the absence of a partner resulted in a worse QOL, while “having a partner” potentiated good physical and mental QOL (35).

The unavailability of daily access to drinking water violates the Human Right to Adequate Food, instituted in Brazil and ensured among the social rights in the Federal Constitution, through the approval of Constitutional Amendment No. 64 (36) The lack of basic sanitation hinders the reduction of infectious diseases and consequently the reduction of FI (37), which can further compromise the tight budget of the low-income population when trying to meet this basic need. The association found in our study between not drinking treated water at home and low perception of QoL may be related to the lack of access to the water supply network, low financial condition, and/or low education, which may lead to the difficulty in understanding information about measures to sanitize the water supply, such as boiling, or adding hypochlorite.

Another point of reflection is about self-reported health conditions. Self-reported morbidities are related to access to health services, which is higher in individuals with better socioeconomic status and lower in more vulnerable people, with lower education and lower income (38). Although Brazil has a Brazilian Public Health System (SUS), which guarantees access to health services by the population with less education, lower income and without health insurance (39), this population may have greater difficulty in receiving medical care, diagnosis and access to medical consultations. This may be due to the insufficient or non-existent offer of consultations in some places, lower availability and offer of services and procedures than users of health plans, less clarification on the importance of access to these services and less financial availability to seek care to private health assistance when they cannot obtain assistance from the public health system (39, 40). On the other hand, the self-reported health condition has been widely used to understand the health of the adult and elderly population, being applied and validated in several Brazilian national studies, such as the National Health Survey (Pesquisa Nacional de Saúde in Portuguese) (41) and the annual surveys on chronic diseases by the Health System. Surveillance of Risk and Protective Factors for Chronic Diseases by Telephone Survey (Vigitel in Portuguese) (42).

This study has limitations. One of them refers to its design (cross-sectional) which does not allow explaining or determining causal pathways between food security status, QoL, and associated factors. Another limiting factor was that, due to the sample size, it was not possible to stratify into groups of adults and older adults. It is understood that aging is a factor generally associated with a low perception of quality of life, especially in non-developed or developing countries, due to the presence of physical limitations and diseases, especially NCDs (25, 43–45). In addition, the type of living arrangement in which the older adults live, usually with sons and/or spouse or alone, favors a low perception of quality of life in this population (46). On the other hand, adults may be more vulnerable to food insecurity as they are mostly the economically active group of the population that are most subject to unemployment or informal employment. In this sense, it can limit access to income (18, 19). Thus, we understand that the associated factors may not be the same as those found in this study.

Despite these limitations, this article has as its strengths the solid methodological basis, derived from a household survey. In addition, the study, unlike other Brazilian studies such as the National Health Survey, considered the inclusion of information on food and nutrition security and the possibility of relating it to important aspects of health and nutrition in a population in the Northeast Brazilian.

We also highlight the importance of quality of life studies in a population-based sample, which can help health professionals in decision-making, given the lack of studies aimed at a comprehensive assessment of QOL in adults and elderly people in situations of food insecurity, especially in a vulnerable socio-environmental region (47).

## Conclusion

Our findings revealed that, for both the FS and the FI groups, the low perception of QoL was associated with the occurrence of NCDs and their risk factors, especially emotional disorders.

It is worth mentioning that, for food insecure individuals, besides the association with NCD/risk factors and emotional disorders, indicators that reflect emotional and/or social support, or those related to basic life needs, such as drinking water, had a risk association for low perceived QoL, further highlighting the social vulnerability of this population group.

It is observed that the results of this study in the field of public health apply to the planning of public policies that aim not only to face food insecurity, the fight against hunger, nutritional deficiencies, and health inequities, but also to address the formation and propagation of healthy food systems, the promotion of physical and mental health, aiming at the reduction of NCDs.

## Data availability statement

The raw data supporting the conclusions of this article will be made available by the authors, without undue reservation.

## Ethics statement

The studies involving human participants were reviewed and approved by Research Ethics Committee of the Onofre Lopes University Hospital of Federal University of Rio Grande do Norte. The patients/participants provided their written informed consent to participate in this study.

## Author contributions

NP contributed to the conception and design, analysis and interpretation of data, elaboration, writing and review of the article. NC contributed to data analysis and review of the article. ÂO participated in the study planning, data analysis, and review of the article. DM, SC, and SL participated in the conception, planning, and review of the study. CR participated in the review of the study. CL participated in the conception and planning of the study, analysis and interpretation of data, elaboration and review of the study. All authors approved the final version of the manuscript.

## Funding

This study was funded in part by the Coordenação de Aperfeiçoamento de Pessoal de Nível Superior (CAPES)—financing code 001 and by the Conselho Nacional de Desenvolvimento Científico e Tecnológico (CNPq)—Grant Number: 431053/2016-2.

## Conflict of interest

The authors declare that the research was conducted in the absence of any commercial or financial relationships that could be construed as a potential conflict of interest.

## Publisher's note

All claims expressed in this article are solely those of the authors and do not necessarily represent those of their affiliated organizations, or those of the publisher, the editors and the reviewers. Any product that may be evaluated in this article, or claim that may be made by its manufacturer, is not guaranteed or endorsed by the publisher.

## References

[1] Brasil. Presidência da República. Lei n° 11.346, de 15 de setembro de 2006. LOSAN - Lei Orgânica de Segurança Alimentar e Nutricional (2006). Available online at: http://www.planalto.gov.br/ccivil_03/_ato2004-2006/2006/lei/l11346.htm (acessed June 02, 2022).

[2] Swinburn B Kraak VI Allender S Atkins VJ Baker PI Bogard JR. The global syndemic of obesity, undernutrition, and climate change: the lancet commission report. Lancet. (2019) 393:791–846. 10.1016/S0140-6736(18)32822-8 30700377

[3] FAO. Q&A: COVID-19 Pandemic – Impact on Food and Agriculture | FAO | Food and Agriculture Organization of the United Nations (2020). Available online at: https://www.fao.org/2019-ncov/q-and-a/en/ (accessed January 13, 2021).

[4] Bezerra TA Olinda RA Pedraza DF. Insegurança alimentar no Brasil segundo diferentes cenários sociodemográficos. Ciência e saúde coletiva. (2017) 22:637–51. 10.1590/1413-81232017222.19952015

[5] Pérez-Escamilla R. Food security and the 2015–2030 sustainable development goals: from human to planetary health. Curr Dev Nutr. (2017) 1:e000513. 10.3945/cdn.117.000513 29955711 PMC5998358

[6] Laraia BA. Food insecurity and chronic disease. Adv Nutr. (2013) 4:203–12. 10.3945/an.112.003277 23493536 PMC3649100

[7] De Oliveira MRM Lima RSDS Da Silva FR Pinto LMO Sampaio RMM. Food and nutrition insecurity and risk factors for chronic noncommunicable diseases among solid waste collectors. DEMETRA Aliment Nutr Saúde. (2018) 13:635–47. 10.12957/demetra.2018.34088

[8] Chung HK Kim OY Kwak SY Cho Y Lee KW Shin MJ. Household food insecurity is associated with adverse mental health indicators and lower quality of life among koreans: results from the Korea national health and nutrition examination survey 2012–2013. Nutrients. (2016) 8:1–13. 10.3390/nu8120819 27999277 PMC5188472

[9] Willem K The The WHOQOL Group. The World Health Organization Quality of Life Assessment (WHQOL): position paper from the World Helth Organization. Soc Sci Med. (1995) 41:1403–9. 10.1016/0277-9536(95)00112-K 8560308

[10] Kihlström L Burris M Dobbins J McGrath E Renda A Cordier T. Food insecurity and health-related quality of life: a cross-sectional analysis of older adults in Florida, U.S. Ecol Food Nutr. (2019) 58:45–65. 10.1080/03670244.2018.1559160 30582362

[11] IBGE. Pesquisa de Orçamentos Familiares 2017-2018. Análise da segurança alimentar no Brasil. Rio de Janeiro: IBGE (2020). 69 p.

[12] Maciel NM Souza MH Conti D Almeida SF Simeão P Vitor C. Sociodemographic factors, level of physical activity and health-related quality of life in adults from the north-east of São Paulo, Brazil: a cross-sectional population study. BMJ Open. (2018) 8:17804. 10.1136/bmjopen-2017-017804 29317412 PMC5780712

[13] BRASIL MDS. Escala Brasileira de Insegurança Alimentar-EBIA: análise psicométrica de uma dimensão da Segurança Alimentar e Nutricional. Estudo Técnico No. 01. Brasília: SAGI (2014). 15 p.

[14] Fleck MPA Louzada S Xavier M Chachamovich E Vieira G Santos L. Aplicação da versão em português do instrumento abreviado de avaliação da qualidade de vida “WHOQOL-bref”. Rev Saude Publica. (2000) 34:178–83. 10.1590/S0034-89102000000200012 10881154

[15] Matsudo S Araújo T Matsudo V Andrade D Andrade E Oliveira LC. Questionário Internacional de Atividade Física (IPAQ): estudo de validade e reprodutibilidade no Brasil. Atividade Fí*sica Saúde*. (2001) 6:1–14. 10.12820/rbafs.v.6n2p5-18

[16] WHO Expert Committee on Physical Status: The Use and Interpretation of Anthropometry: Report of a WHO Expert Committee - WHO Techical Report Series 854. Switzerland: Benteli (1995). 452 p. 8594834

[17] Lipschitz DA. Screening for nutritional status in the elderly. Prim Care. (1994) 21:55–67. 10.1016/S0095-4543(21)00452-8 8197257

[18] Santos TG Silveira JAC Longo-Silva G Ramires EKNM Menezes RCE. Tendência e fatores associados à insegurança alimentar no Brasil: Pesquisa Nacional por Amostra de Domicílios 2004, 2009 e 2013. Cad Saúde Pública. (2018) 34:e00066917. 10.1590/0102-311x00066917 29617484

[19] Morais DC Lopes SO Priore SE. Indicadores de avaliação da Insegurança Alimentar e Nutricional e fatores associados: revisão sistemática. Ciênc saúde coletiva. (2020) 25 :2687–700. 10.1590/1413-81232020257.23672018 32667551

[20] Bezerra MS Jacob MCM Ferreira MAF Vale D Mirabal IRB Lyra CO. Insegurança alimentar e nutricional no Brasil e sua correlação com indicadores de vulnerabilidade. Ciênc saúde coletiva. (2020) 25 :3833–46. 10.1590/1413-812320202510.35882018 32997016

[21] Russel JC Flood V Yeatman H Wang J Mitchell P. Food insecurity and poor diet quality are associated with reduced quality of life in older adults. Nutr Diet. (2016) 73:50–8. 10.1111/1747-0080.12263

[22] Gregório MJ Rodrigues AM Graça P de Sousa RD Dias SS Branco JC. Food insecurity is associated with low adherence to the mediterranean diet and adverse health conditions in Portuguese adults. Front Public Health. (2018) 6:38. 10.3389/fpubh.2018.00038 29515992 PMC5826370

[23] Noronha DD Martins AMEBL Dias DS Silveira MF De Paula AMB Haikal DSA. Qualidade de vida relacionada à saúde entre adultos e fatores associados: um estudo de base populacional. Cien Saude Colet. (2016) 21:463–74. 10.1590/1413-81232015212.01102015 26910154

[24] Chung J Han C. Health related quality of life in relation to asthma – data from a cross sectional study. J Asthma. (2017) 4:1–7. 10.1080/02770903.2017.1387266 28976222

[25] Lodhi FS Montazeri A Nedjat S Mahmoodi M Farooq U Yaseri M. Assessing the quality of life among Pakistani general population and their associated factors by using the World Health Organization's quality of life instrument (WHOQOL-BREF): a population based cross-sectional study. Health Qual Life Outcomes. (2019) 17:9. 10.1186/s12955-018-1065-x 30642360 PMC6332637

[26] Gajda R Jezewska-Zychowicz M. Elderly perception of distance to the grocery store as a reason for feeling food insecurity-can food policy limit this? Nutrients. (2020) 12:3191. 10.3390/nu12103191 33086560 PMC7603094

[27] Rong J CHEN G WANG X GE Y MENG N XIE T. Correlation between depressive symptoms and quality of life, and associated factors for depressive symptoms among rural elderly in Anhui, China. Clin Interv Aging. (2019) 14:1901–10. 10.2147/CIA.S225141 31806946 PMC6839580

[28] Nagata JM Palar K Gooding HC Garber AK Whittle HJ Bibbins-Domingo K. Food insecurity is associated with poorer mental health and sleep outcomes in young adults. J Adolesc Heal. (2019) 65:805–11. 10.1016/j.jadohealth.2019.08.010 31587956 PMC6874757

[29] Academy Academy of Sleep Medicine A Research Society S. Joint consensus statement of the american academy of sleep medicine and sleep research society on the recommended amount of sleep for a healthy adult: methodology and discussion. J Clin Sleep Med. (2015) 11:931–52. 10.5664/jcsm.4950 26235159 PMC4513271

[30] Lee YS Kim TH. Household food insecurity and breakfast skipping: their association with depressive symptoms. Psychiatry Res. (2019) 271:83–8. 10.1016/j.psychres.2018.11.031 30471489

[31] Barros MB. Guimarães Lima M Ceolim MF Zancanella E Cardoso TAM. Quality of sleep, health and well-being in a population-based study. Rev Saude Publica. (2019) 53:82. 10.11606/s1518-8787.2019053001067 31576942 PMC6763282

[32] Uchmanowicz I Markiewicz K Uchmanowicz B Kołtuniuk A Rosińczuk J. The relationship between sleep disturbances and quality of life in elderly patients with hypertension. Clin Interv Aging. (2019) 14:155–65. 10.2147/CIA.S188499 30697040 PMC6339653

[33] Min J Zhao Y Slivka L Wang Y Fisher J. Double burden of diseases worldwide: coexistence of undernutrition and over-nutrition-related non-communicable chronic diseases. Obes Ver. (2018) 19:49–61. 10.1111/obr.12605 28940822 PMC5962023

[34] Jj Gobbens R Remmen R. The effects of sociodemographic factors on quality of life among people aged 50 years or older are not unequivocal: comparing sF-12, WhOQOl-BreF, and WhOQOl-OlD. Clin Interv Aging. (2019) 14:231–9. 10.2147/CIA.S189560 30787599 PMC6363394

[35] Santos Campos M Flor L. Fatores associados à qualidade de vida de brasileiros e de diabéticos: evidências de um inquérito de base populacional. Cien Saude Colet. (2019) 24:1007–20. 10.1590/1413-81232018243.09462017 30892521

[36] Brasil Presidência da República,. Emenda Constitucional n^*o*^ *64, de 04 de fevereiro de 2010* (2010). Available online at: http://www.planalto.gov.br/ccivil_03/constituicao/emendas/emc/emc64.htm (accessed June 13, 2021).

[37] Nações unidas,. Transformando Nosso Mundo: A Agenda 2030 para o Desenvolvimento Sustentável (2015). Available online at: http://www.nacoesunidas.org/wp-content/uploads/2015/10/agenda2030-pt-br.pdf (accessed February 26, 2021).

[38] Malta DC Bernal RTI Gomez CS Cardoso LSM Lima MG Barros MBA. Inequalities in the use of health services by adults and elderly people with and without noncommunicable diseases in Brazil, 2019 National Health Survey. Rev Bras Epidemiol. (2021) 24:e210003. 10.1590/1980-549720210003.supl.2 34910057

[39] Malta DC Bernal RTI Lima MG Araújo SSC Silva MMA Freitas MIF. Noncommunicable diseases and the use of health services: analysis of the National Health Survey in Brazil. Rev Saude Publica. (2017) 51:4s. 10.1590/s1518-8787.2017051000090 28591353 PMC5676356

[40] Silva CR Carvalho BG Júnior LC Almeida Nunes EFP. Difficulties in accessing services that are of medium complexity in small municipalities: a case study. Cien Saude Colet. (2017) 22:1109–20. 10.1590/1413-81232017224.27002016 28444038

[41] Brasil. Ministério do Planejamento, orçamento e gestão. Instituto Brasileiro de Geografia e Estatística. Pesquisa Nacional de Saúde: 2013: acesso e utilização dos serviços de saúde, acidentes e violências: Brasil, grandes regiões e unidades da federação / IBGE, Coordenação de Trabalho e Rendimento. – Rio de Janeiro: IBGE (2015). 100 p.

[42] Brasil. Ministério da Saúde. Secretaria de Vigilância em Saúde. Departamento de Análise em Saúde e Vigilância de Doenças Não Transmissíveis. Vigitel Brasil 2006-2021: vigilância de fatores de risco e proteção para doenças crônicas por inquérito telefônico: estimativas sobre frequência e distribuição sociodemográfica de morbidade referida e autoavaliação de saúde nas capitais dos 26 estados brasileiros e no Distrito Federal entre 2006 e 2021: morbidade referida e autoavaliação de saúde [recurso eletrônico] / Ministério da Saúde, Secretaria de Vigilância em Saúde, Departamento de Análise em Saúde e Vigilância de Doenças Não Transmissíveis. – Brasília: Ministério da Saúde (2022). 55 p.

[43] Tourani S Behzadifar M Martini M Aryankhesal A Mirghaed MT Salemi M. Health-related quality of life among healthy elderly Iranians: a systematic review and meta-analysis of the literature. Health Qual Life Outcomes. (2018) 16:1–9. 10.1186/s12955-018-0845-7 29347951 PMC5774099

[44] Wong ELY Xu RH Cheung AWL. Health-related quality of life among patients with hypertension: population-based survey using EQ5D-5L in Hong Kong SAR, China. BMJ Open. (2019) 9:32544. 10.1136/bmjopen-2019-032544 31562165 PMC6773333

[45] Liang Z Zhang T Lin T Liu L Wang B Fu A Z. Health-related quality of life among rural men and women with hypertension: assessment by the EQ-5D-5L in Jiangsu, China. Qual Life Res. (2019) 28:2069–80. 10.1007/s11136-019-02139-3 30830645

[46] Bolina AF Araújo MC Hass VJ Tavares DMS. Association between living arrangement and quality of life for older adults in the community. Rev Latino Am Enfermagem. (2021) 29:e3401. 10.1590/1518-8345.4051.3401 33439953 PMC7798390

[47] Pequeno NPF de Araújo Cabral NL Marchioni DM Lima SCVC de Oliveira Lyra C. Quality of life assessment instruments for adults: a systematic review of population-based studies. Health Qual Life Outcomes. (2020) 18:1–13. 10.1186/s12955-020-01347-7 32605649 PMC7329518

